# Demographic and clinical characteristics associated with screening practices for hydroxychloroquine retinopathy

**DOI:** 10.1038/s41598-024-51667-7

**Published:** 2024-01-10

**Authors:** Jiyeong Kim, Hyo Chan Jeong, Hyeon Yoon Kwon, Young Hwan Kim, Seong Joon Ahn

**Affiliations:** 1 Department of Pre-Medicine, College of Medicine, and Biostatistics Lab, Medical Research Collaborating Center, Hanyang University, Seoul, Republic of Korea; 2 Department of Ophthalmology, Hanyang University Hospital, Hanyang University College of Medicine, 222-1 Wangsimni-ro, Seongdong-gu, Seoul, 04763 Republic of Korea

**Keywords:** Eye diseases, Retinal diseases

## Abstract

In this nationwide population-based cohort study, we investigated the demographic and clinical characteristics associated with hydroxychloroquine retinopathy screening using the National Health Insurance Review and Assessment database in South Korea. This study included a total of 32,732 at-risk patients, identified based on having been prescribed hydroxychloroquine for at least 6 months, and 15,477 long-term (> 5 years) users between January 2010 and December 2020. Participants were categorized based on the performance of baseline examinations (within 1 year of hydroxychloroquine use) and monitoring examinations (after 5 years of hydroxychloroquine use). Demographic and clinical factors, including hospitals and medical specialties prescribing hydroxychloroquine, indications for hydroxychloroquine use, and prescription details, were compared between groups. Significant differences were found in sex, residence, departments and hospitals (primary vs. referral centers) where hydroxychloroquine was prescribed, diagnosis for hydroxychloroquine therapy, and mean daily dose between patients who did and did not undergo baseline or monitoring examinations (all P < 0.01). Patients who received hydroxychloroquine prescriptions from referral hospitals were more likely to undergo baseline and monitoring examinations compared to those from primary clinics (both P < 0.001). Additionally, patients who received hydroxychloroquine prescriptions from the rheumatology department and had systemic lupus erythematosus were more likely to undergo baseline and monitoring examinations compared to other patients (all P < 0.001). There were notable differences in the number of modalities used for retinopathy screening between primary and referral centers (P < 0.001). Our findings suggest that several clinical factors related to hydroxychloroquine prescription and screening centers are associated with retinopathy screening practices.

## Introduction

Hydroxychloroquine (HCQ) is widely prescribed for the treatment of several rheumatologic and dermatologic diseases, such as systemic lupus erythematosus (SLE) and rheumatoid arthritis (RA)^1,2^. HCQ retinopathy, a relatively rare but potentially serious side effect, is a well-known drug-induced retinal toxicity. It is characterized by possibly irreversible and progressive defects in the outer retinal layers, involving the photoreceptors or retinal pigment epithelium, and potentially leading to permanent visual impairment. The prevalence has recently been estimated at 5–10% among long-term (5 or more years) HCQ users, which is higher than that in previous reports^3–6^. Modern imaging modalities such as optical coherence tomography (OCT) and fundus autofluorescence (FAF) enable better and earlier detection of retinopathy by providing objective evidence on retinal changes caused by the toxicity^6–8^. Further, national guidelines such as American Academy of Ophthalmology (AAO) and Royal College of Ophthalmologists (RCOphth) recommendations have been published and implemented for HCQ retinopathy screening worldwide^8,9^.

Despite technological advances and organizational efforts, late diagnosis is a significant problem in HCQ retinopathy^6,8,10–14^. This is critical, as eyes with more advanced stages show continuous progression even after drug cessation in previous studies, leading to fovea-threatening disease and progressive vision loss^3–6^. Therefore, to minimize irreversible, progressive vision loss and structural damage, screening practices should be performed appropriately in accordance with current guidelines.

Few studies have been conducted on real-world screening practices. For example, Au et al. reported that appropriate screening was performed in approximately half of the patients within a large multispecialty ophthalmic practice of a single institution^15^. A population-based study performed in Taiwan revealed that the percentages of patients receiving screening examination were around 1%^16^. These low proportions receiving proper screening raise significant concerns on the appropriateness of real-world screening practices. Furthermore, the factors influencing practice patterns should be investigated to identify the target population for directing efforts or guiding clinicians to better implement the current recommendations.

Accordingly, the present study aimed to investigate demographic and clinical characteristics associated with HCQ retinopathy screening practice patterns in a nationwide population-based cohort in Korea. By reflecting real-world practice patterns across all primary/tertiary centers at the national level, this study addressed the factors associated with practice patterns and the factors that should be considered to enhance adherence.

## Methods

### Subjects

South Korea has a mandatory universal health insurance system that provides medical care to almost its entire population (97% of the South Korean population) and all prescriptions and procedures (examinations) are recorded in the national Health Insurance Review and Assessment (HIRA) database, which has been described in detail in previous studies^17–19^. This nationwide, population-based study used data from the HIRA database recorded during the period from January 1, 2007 to December 31, 2021. Patients taking HCQ were identified by searching those with the drug component codes for HCQ (171701ATB, 171702ATB, 171703ATB, 171704ATB, or 171705ATB). Subjects at risk for retinopathy and those taking HCQ for 6 months or longer^18^ were included in this study.

**Figure 1:**
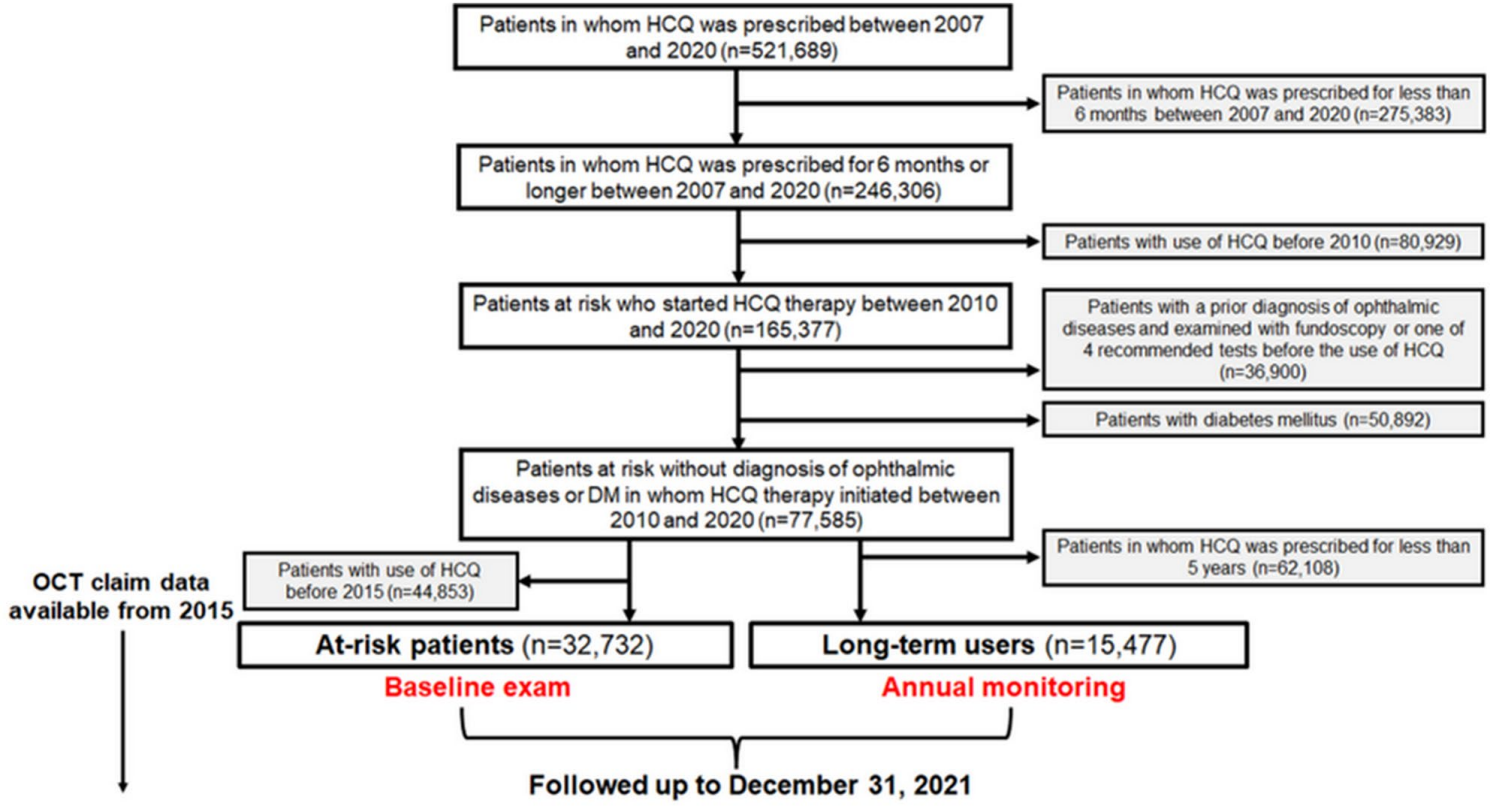
A flowchart of the study population and inclusion/exclusion criteria used.

To ensure a precise estimation of the duration of HCQ use, we excluded patients who had received HCQ between January 1, 2007, and December 31, 2009. This is because these individuals might have been prescribed HCQ prior to the start date of the inclusion period (January 1, 2007), making an accurate estimation of the duration unattainable. To exclude patients with prior ophthalmic diseases requiring follow-up examinations during the observation period, we excluded those examined with fundoscopy or any of the four screening modalities recommended by the AAO and those diagnosed with ophthalmic diseases before the use of HCQ. Furthermore, those diagnosed with diabetes mellitus (DM) between January 1, 2007 and the end of the study date (December 31, 2021) were excluded as retinopathy screening, if done, could be performed for diabetic retinopathy. Accordingly, at-risk patients without prior ophthalmic disease or DM (requiring follow-up ophthalmic examinations or regular retinopathy screening) who started HCQ therapy in 2010 were included in this study. A flowchart indicating the inclusion and exclusion criteria and the number of patients meeting the criteria is presented in Fig. 1. This study was approved by the Institutional Review Board of Hanyang University Hospital (IRB File ID: 2022-07-039) and was conducted in accordance with the principles of the Declaration of Helsinki. The need for informed consent was waived by the Institutional Review Board of Hanyang University Hospital because of the retrospective nature of the study and the use of deidentified data.

### Definitions

The most recent AAO guidelines (2016) recommend two types of screening examinations: baseline examination and annual screening. For the baseline examination, the guidelines recommend that all patients beginning long-term HCQ therapy should undergo a baseline ophthalmologic examination within the first year of starting the drug. However, the definition of ‘long-term’ is not specified, and no consensus regarding the target population (namely, the minimum period of HCQ exposure for screening) has been reached. However, the guidelines also suggest that annual screening can be deferred until there have been 5 years of exposure^8,9^.

Accordingly, two target populations for baseline examination and annual monitoring were defined: the population at risk of retinopathy (thus subject to baseline screening) and those requiring annual monitoring. As in our previous study, the ‘at-risk’ patients and ‘long-term’ users were defined as those who used HCQ for ≥ 6 months and ≥ 5 years, respectively^18^. In this study, we used total prescription days to represent the duration of HCQ use (exposure) to minimize the effect of gaps between one prescription and another^18^.

We defined baseline screening as the first ophthalmic examination performed for at-risk patients within 1 year of HCQ initiation using fundoscopy/fundus photography or any of the four standard tests recommended by the AAO (OCT, FAF, automated visual field [VF], and multifocal electroretinogram [mfERG]) and monitoring examinations as those performed after 5 years of use for long-term users using any of the four standard modalities, as per the most recent AAO guidelines^8^.

### Evaluations

The timing and modalities used for baseline screening and annual monitoring were evaluated. However, as health claims data on OCT became available in the HIRA database from January 1, 2015, the performance and number of screening tests performed for baseline and monitoring examinations could be assessed correctly from January 1, 2015. Therefore, our analyses utilized data from baseline screening and monitoring examinations obtained from January 1, 2015 to December 31, 2021.

The patients were separated into subgroups based on baseline performance and monitoring examinations. Patients were also divided into subgroups based on the number of modalities used for monitoring examinations (single or multiple modalities). Demographic and clinical characteristics were compared between patients with and without baseline examinations, and between those with and without monitoring. The demographic and clinical characteristics for comparative analyses included age, sex, residence, medical specialties prescribing HCQ, hospitals (level of care) of prescription, hospitals for retinopathy screening, medical indications for HCQ use, daily dose, and duration of HCQ use.

### Analyses

Categorical variables were recorded as frequency and percentage, and continuous variables as median (Q1 [first quartile]–Q3 [third one]) or mean ± standard deviation. The chi-squared test was used to compare categorical variables between the groups. Multivariate logistic regression was performed to identify demographic and clinical factors associated with baseline performance and monitoring examinations. Statistical significance was determined using a two-sided test with a significance level of 0.05. SAS Enterprise Guide 7.1 (SAS Institute, Cary, NC) was used for all analyses.

## Results

### Demographic and clinical characteristics

A total of 32,732 at-risk patients and 15,477 long-term users were included in the analyses (Table 1). Most patients were women, comprising 79.4% and 85.9% of at-risk patients and long-term users, respectively. The age distributions of the two cohorts are presented in Table 1; the most common age group was between 50 and 59 years, consisting of 27.1% and 25.9% of the overall and long-term users, respectively. Patients with RA comprised approximately two-thirds of the patients, and rheumatologists were the most common prescribing physicians, accounting for 65.0% and 60.2% of the overall and long-term users, respectively. The mean duration of HCQ use was 29.5 ± 20.0 and 93.0 ± 21.2 months for overall and long-term users, respectively.

**Table 1:** Demographic and clinical information of overall hydroxychloroquine (HCQ) users and long-term (≥ 5 years) users.

Characteristics	Overall users (n = 32,732)	Long-term users (n = 15,477)
Age, mean (years)	47.4 ± 15.0	45.6 ± 14.4
< 30	4296 (13.1%)	2277 (14.7%)
30–39	5224 (16.0%)	2741 (17.7%)
40–49	7760 (23.7%)	3945 (25.5%)
50–59	8871 (27.1%)	4015 (25.9%)
≥ 60	6581 (20.1%)	2499 (16.1%)
Sex		
Male:female	6742:25,990 (20.6%:79.4%)	2183:13,294 (14.1%:85.9%)
Residence		
Metropolitan area/large cities:small cities/rural	16,610:16,122 (50.8%:49.3%)	7442:8035 (48.1%:51.9%)
Medical specialties prescribing HCQ		
Rheumatology	19,213 (58.7%)	9299 (60.1%)
Dermatology	1165 (3.6%)	336 (2.2%)
Internal medicine other than rheumatology	9183 (28.1%)	4403 (28.5%)
Others	3171 (9.7%)	1439 (9.3%)
Hospitals of prescription		
Primary:referral centers	7325:25,407 (22.4%:77.6%)	2801:12,676 (18.1%:81.9%)
Medical indications for HCQ use		
SLE:RA:others	5862:21,264:5606 (17.9%:65.0%:17.1%)	3417:9319:2741 (22.1%:60.2%:17.7%)
Mean duration (months)	29.5 ± 20.0	93.0 ± 21.2
Daily dose, mean (mg)	250.1 ± 80.9	257.6 ± 75.7
< 200: 200–299: ≥ 300	6713:15,962:10,057 (20.5:48.8:30.7%)	3464:7380:4633 (22.4:47.7:29.9%)

*SLE* systemic lupus erythematosus, *RA* rheumatoid arthritis.

### Characteristics associated with performance of baseline examination and monitoring

**Figure 2:**
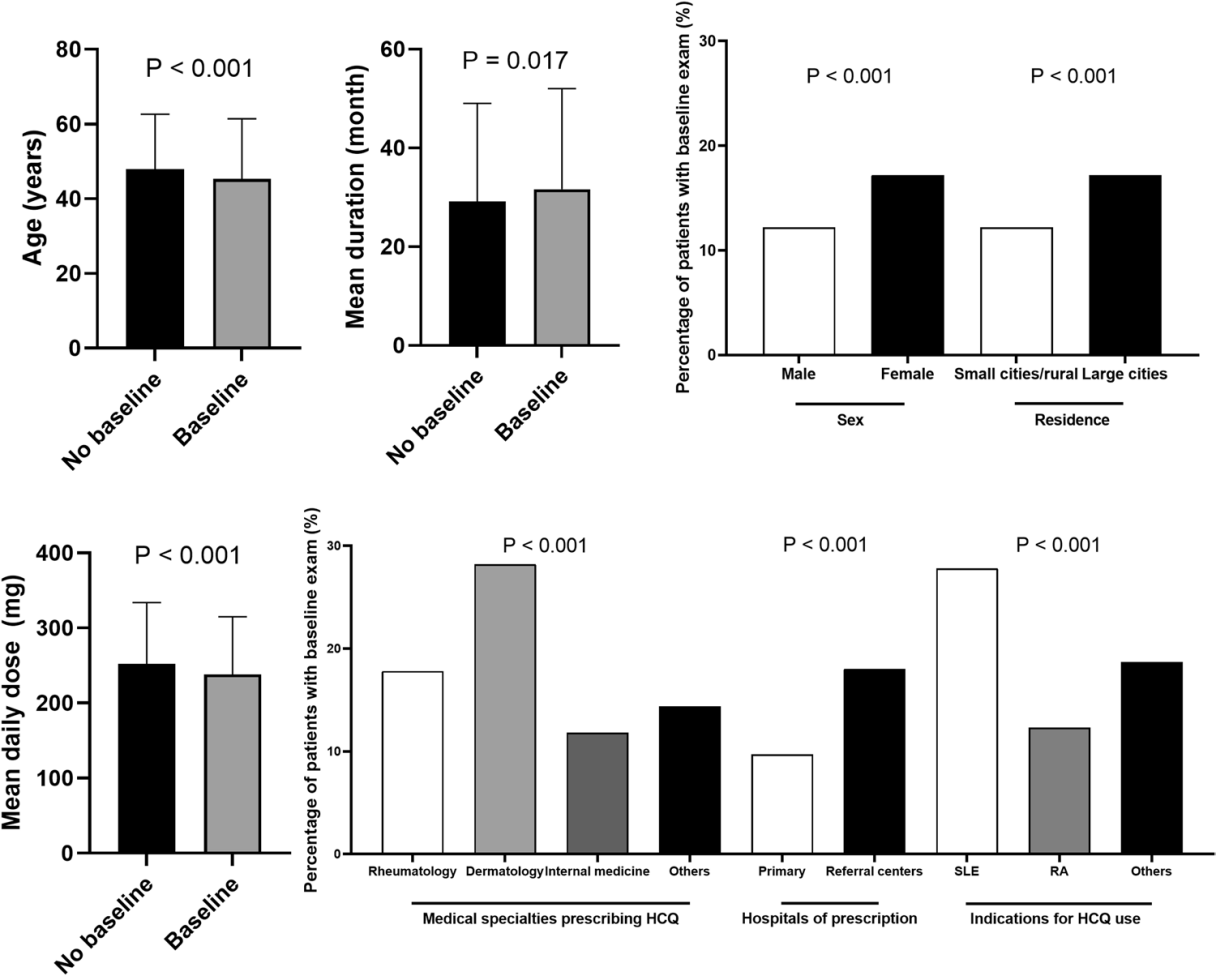
Comparing demographic and clinical characteristics between patients who underwent baseline examination within 1 year of hydroxychloroquine initiation and those who did not. *HCQ* hydroxychloroquine, *SLE* systemic lupus erythematosus, *RA* rheumatoid arthritis.

Baseline screening was performed within 1 year of HCQ use in 5287 of the 32,732 (16.2%) at-risk patients in our cohort. Figure 2 presents a comparison of demographic and clinical characteristics between patients with and without baseline examinations performed within 1 year of HCQ use. There were significant differences in age, sex, residence, medical specialties prescribing the drug, hospital (level of care) of prescription, indications for HCQ use, mean daily dose, and mean duration of HCQ use between patients with and without baseline examination (all *P* < 0.05).

**Figure 3:**
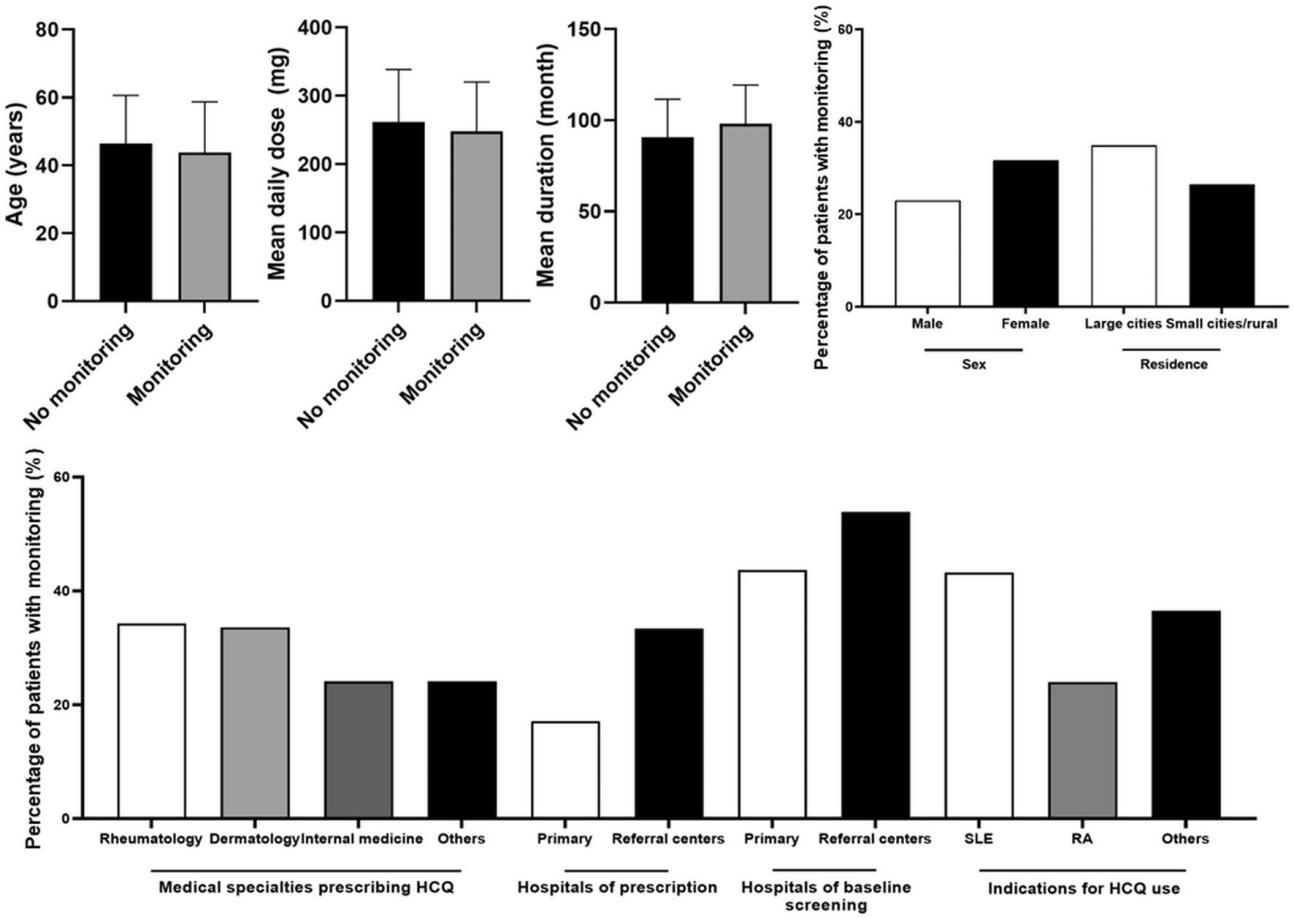
Comparison of demographic and clinical characteristics between monitored and non-monitored long-term users. Statistically significant differences (P < 0.001) were observed across all compared items. *HCQ* hydroxychloroquine, *SLE* systemic lupus erythematosus, *RA* rheumatoid arthritis.

Monitoring examinations were performed after 5 years of use in 4711 of the 15,477 (30.4%) long-term users. In the comparison of demographic and clinical characteristics between those with and without monitoring examinations (Fig. 3), there were significant differences in mean age, sex, residence, medical specialties prescribing HCQ, hospitals of prescription, indications for HCQ use, mean daily dose, and mean duration of HCQ use (all *P* < 0.001). Specifically, young females, those living in metropolitan areas or large cities, and those receiving HCQ from rheumatologists, referral centers, or those with SLE were more likely to receive monitoring examinations. Those who underwent monitoring examinations after 5 years of use had a smaller mean daily dose and longer duration of HCQ use than those who did not. Interestingly, those who underwent baseline examinations at referral centers were more likely to undergo monitoring examinations. Supplementary Table S1 further validates the significant association between baseline and monitoring examinations in hospitals (level of care) of screening, as both baseline and monitoring examinations were likely performed at the same level of care (primary vs. referral centers; *P* < 0.001).

### Characteristics associated with modalities of screening examinations

Among patients receiving baseline examination within 1 year of HCQ use, there were significant differences in age, residence, medical specialties prescribed HCQ, hospitals of prescription, indication of HCQ use, mean duration of HCQ therapy, and mean daily dose between patients with and without additional modalities (other than fundoscopy or fundus photography) performed, OCT and/or automated VF (all *P* < 0.001; Supplementary Table S2).

For monitoring examinations, 1 and 2 or more modalities were used in 1593 (33.8%) and 3118 (66.2%) patients, respectively. Table 2 shows a comparison of demographic and clinical characteristics between patients who underwent single and multiple screening modalities. There were significant differences in age, residence, medical specialties prescribing HCQ, hospitals of prescription and monitoring, indications for HCQ use, mean duration, and mean daily dose between groups (all *P* < 0.05). For example, multiple modalities were more frequently used for retinopathy screening in female patients; those in metropolitan areas or large cities; and those prescribed HCQ by rheumatology, referral centers, or for SLE. Patients with a single modality had significantly higher mean daily doses than those with multiple modalities (*P* < 0.001). In particular, there were remarkable differences in the number of modalities used according to the monitoring hospitals, as retinopathy screening was conducted using two or more modalities in 44.3% of patients at primary hospitals and 79.1% of patients at referral hospitals. Supplementary Table S3 illustrates the utilization patterns of the four screening modalities recommended by the 2016 AAO guidelines for retinopathy monitoring in both the overall monitored patient cohort and specific residence and hospital subgroups, potentially reflecting variations in the availability of FAF.

**Table 2:** Number of modalities used and their association with demographic and clinical characteristics in the monitored population.

Characteristics	1 (n = 1593)	2 or more (n = 3118)	P-value
Mean age (years)	49.0 ± 14.0	41.1 ± 14.6	< 0.001
Sex			
Male	173 (34.5%)	328 (65.5%)	0.720
Female	1420 (33.7%)	2790 (66.3%)	
Residence			
Metropolitan area/large cities	704 (27.1%)	1890 (72.9%)	< 0.001
Small cities/rural	889 (42.0%)	1228 (58.0%)	
Medical specialties prescribing HCQ			
Rheumatology	996 (31.2%)	2194 (68.8%)	< 0.001
Dermatology	25 (22.1%)	88 (77.9%)	
Internal medicine other than rheumatology	443 (41.7%)	618 (58.3%)	
Others	129 (37.2%)	218 (62.8%)	
Hospitals of prescription			
Primary	285 (59.5%)	194 (40.5%)	< 0.001
Referral centers	1,308 (30.9%)	2,924 (69.1%)	
Hospitals of monitoring			
Primary	975 (55.7%)	775 (44.3%)	< 0.001
Secondary/tertiary	618 (20.9%)	2,343 (79.1%)	
Indications for HCQ use			
SLE	295 (20.0%)	1182 (80.0%)	< 0.001
RA	976 (43.7%)	1258 (56.3%)	
Others	322 (32.2%)	678 (67.8%)	
Mean duration (month)	96.9 ± 21.5	98.5 ± 21.2	0.017
Mean daily dose (mg)	254.5 ± 75.3	244.4 ± 70.7	< 0.001

Percentages indicate the distribution of each characteristic between the two groups of modalities used.

*HCQ* hydroxychloroquine, *SLE* systemic lupus erythematosus, *RA* rheumatoid arthritis.

Among the 4711 patients receiving a monitoring examination, a second monitoring examination was performed in 991 (21.0%). Supplementary Table S4 shows the interval between the first and second monitoring examinations and a comparison of the demographic and clinical characteristics between subgroups separated based on the interval. The second monitoring examination was performed in 422 (42.6%), 449 (45.3%), and 120 (12.1%) patients within 1 year of the first monitoring, between 1 and 2 years after the first, and at an interval of 2 years or longer from the first, respectively. The mean duration of HCQ use were significantly different among the subgroups (both *P* < 0.05).

### Factors associated with performance of baseline and monitoring examinations

Table 3 shows the demographic and clinical characteristics significantly associated with baseline and monitoring examinations in the multivariate logistic regression analyses using stepwise selection. Baseline examination performance was significantly associated with age, sex, residence, hospitals prescribing HCQ, indication for HCQ use, and mean daily dose (all *P* < 0.01). Monitoring was significantly associated with age, sex, residence, medical specialties prescribing HCQ, prescription hospitals, indications for HCQ use, mean duration, and mean daily dose (all *P* < 0.001). Both analyses suggested that medical specialties prescribing HCQ were significantly associated with screening examinations for both baseline and monitoring examinations. However, dermatologists were significantly associated with better baseline screening, whereas rheumatologists were significantly associated with better monitoring examinations.

**Table 3:** Multivariate logistic regression analysis to identify associated factors with baseline and monitoring examinations.

Characteristics	Baseline		Monitoring	
	OR (95% CI)	P-value	OR (95% CI)	P-value
Age (years)	0.997 (0.995–0.999)	0.005	0.989 (0.986–0.993)	< 0.001
Sex				
Male	1 (reference)		1 (reference)	< 0.001
Female	1.314 (1.209–1.428)		1.346 (1.176–1.541)	
Residence				
Metropolitan area/large cities	1 (reference)	0.004	1 (reference)	< 0.001
Small cities/rural	0.912 (0.857–0.971)		0.657 (0.601–0.718)	
Medical specialties prescribing HCQ				
Rheumatology	1.074 (0.961–1.200)	< 0.001	1.185 (1.006–1.396)	< 0.001
Dermatology	1.522(1.281–1.807)		0.994 (0.727–1.358)	
Internal medicine other than rheumatology	0.870 (0.766–0.988)		0.845 (0.707–1.011)	
Others	1 (reference)		1 (reference)	
Hospitals of prescription				
Primary	1 (reference)	< 0.001	1 (reference)	< 0.001
Referral centers	1.563 (1.407–1.736)		2.312 (1.975–2.708)	
Indications for HCQ use				
SLE	1.713 (1.561–1.881)	< 0.001	1.349 (1.190–1.529)	< 0.001
RA	0.689 (0.634–0.748)		0.570 (0.508–0.641)	
Others	1 (reference)		1 (reference)	
Mean duration (month)	1.000 (0.998–1.002)	0.892	1.017 (1.015–1.019)	< 0.001
Mean daily dose (mg)	0.998 (0.998–0.999)	< 0.001	0.998 (0.998–0.999)	< 0.001

*OR* odds ratio, *CI* confidence interval, *HCQ* hydroxychloroquine, *SLE* systemic lupus erythematosus, *RA* rheumatoid arthritis.

## Discussion

National guidelines, such as the 2016 AAO recommendations and the RCOphth guidelines released in 2020, have two key recommendations for retinopathy screening practices: when and how to perform screening for HCQ retinopathy^8,9^. These guidelines address the timing and modalities of appropriate screening for early detection of HCQ toxicity. In a large cohort from a nationwide health claims database in South Korea, the present study addressed the factors associated with each of the two aspects of screening practices. The findings revealed several important factors associated with screening practices for HCQ retinopathy, providing valuable insights for improving screening guidelines and healthcare delivery.

Although national guidelines have been established for HCQ retinopathy screening, appropriate screening as per the guidelines was achieved in a minority of patients taking the drug in prior studies^16^. Our previous study also showed that most patients did not receive monitoring examination after 5 years of use in South Korea^18^, suggesting that late diagnosis, which is a critical issue for HCQ retinopathy screening, may originate from poor adherence to the guidelines. Furthermore, as modalities used for screening varied widely in the study, practices may vary significantly according to countries, regions, and even hospitals (level of care); however, what affects the performance of screening itself and its practice patterns is virtually unknown. This study aimed to address the problems in screening practices performed in South Korea and how these can be improved by a better understanding of the associated factors.

As shown in Figs. 1 and 2, the major problem in HCQ retinopathy screening was unscreened (by baseline or monitoring) patients, consisting of the majority of HCQ users at risk of retinopathy. The performance of screening examinations and the factors influencing their performance are of utmost importance. By dividing the screening examinations into baseline and monitoring examinations, as in the most recent AAO guidelines, we separately analyzed the factors associated with retinopathy screening performance. Interestingly, the clinical factors associated with screening performance were similar between baseline and monitoring examinations, including medical specialties and hospitals prescribing HCQ.

We observed that a substantial proportion of at-risk patients did not undergo baseline screening within the recommended timeframe. Only 16.2% of at-risk patients received baseline examination within 1 year of HCQ use. This finding suggests a potential gap in the delivery of care, as baseline examinations are crucial for establishing a baseline for future comparisons and identifying pre-existing ocular abnormalities. Significant differences were observed between patients with and without baseline examination in terms of age, sex, residence, medical specialties prescribing the drug, hospitals of prescription, indications for HCQ use, mean daily dose, and mean duration of HCQ use. These disparities may reflect variations in healthcare access, awareness, or physician practice patterns. Strategies to improve the uptake of baseline examinations should target these specific patient and healthcare system factors.

Another important finding was the higher percentage of patients receiving HCQ prescriptions from the rheumatology department and of those with SLE in the groups that underwent baseline and monitoring examinations. This finding indicates that patients with rheumatological conditions, particularly those with SLE, are more likely to undergo regular ophthalmic examinations. The heightened awareness of the potential ocular complications associated with HCQ therapy among patients with rheumatological diseases or their healthcare providers (rheumatologists) may contribute to this trend. This suggests a proactive approach in which a higher proportion of patients, due to their condition or their healthcare providers' awareness, either voluntarily visit ophthalmologic clinics or are referred to the ophthalmology department for screening examinations. Furthermore, rheumatologists may also have a higher awareness of the potential ocular complications of autoimmune diseases, such as uveitis, leading to better referrals to ophthalmologists for screening for diseases other than HCQ retinopathy. Nevertheless, our data lacked the specificity to precisely elucidate the factors contributing to this finding, necessitating future investigations to validate and explore the underlying causes of this observed tendency.

Recently, the American College of Rheumatology, AAO, American Academy of Dermatology, and Rheumatologic Dermatology Society provided a joint statement that applies to various medical specialties prescribing drugs and ophthalmologists^20^. Such efforts may enhance the referral of ophthalmologists by common prescribing physicians, rheumatologists, and dermatologists by enhancing awareness and compliance with screening guidelines. This suggests that it is important for healthcare providers, not only ophthalmologists but also prescribing physicians from diverse medical specialties, to be aware of the recommended screening guidelines and to follow them appropriately.

The study also revealed significant differences in the number of modalities used for retinopathy screening between primary and referral centers. This finding suggests that the level of care provided by the screening hospitals plays a role in determining the intensity and comprehensiveness of the screening process. Referral centers may have better access to specialized ophthalmic equipment and expertise, leading to a higher utilization of the recommended screening modalities. In contrast, primary centers may have limited resources and lower awareness of specific screening guidelines, resulting in lower utilization of the recommended modalities.

Our results also raise an important issue regarding the regional disparities in healthcare services. In our study, among the four recommended modalities, FAF exhibited notable variations in utilization between metropolitan areas/large cities and small cities/rural areas (Supplementary Table S3). Differences in access, availability, and quality of healthcare facilities and resources between metropolitan area/large cities and small cities/rural area may lead to regional disparities in screening services for HCQ retinopathy in South Korea, in which medical facilities and professionals are particularly concentrated in the Seoul metropolitan area or large cities. Differences in the socioeconomic status of residents may also result in differences in healthcare access and the number or modality of choice for screening examinations, which are directly related to the cost of screening.

Several limitations of the present study require a careful interpretation of the data. Most importantly, South Korea is different from other countries for several reasons, and these findings may be specific to the South Korean population. The external validity of our findings should be validated in future studies involving other populations. Although consensus over the definitions of at-risk patients and long-term users has not been obtained, we have defined at-risk patients and long-term users as those taking HCQ for ≥ 6 months and ≥ 5 years, respectively. However, this definition, particularly for those at risk, can change if retinal toxicity occurs within six months of use. Additionally, ophthalmic examinations for HCQ screening were specifically identified in the codes (i.e., patients may have undergone ophthalmologic testing for other reasons). Although we excluded patients diagnosed with ophthalmic disease before HCQ therapy and those with DM requiring regular ophthalmic examinations, we cannot completely rule out ophthalmic examinations performed for other purposes. Furthermore, our database lacks information on real body weight and height, which are necessary for calculating the ideal body weight. Consequently, major risk factors such as daily dose/real body weight (as per the 2016 AAO guidelines) and daily dose/ideal body weight (as per the 2011 AAO guidelines) could not be identified in our study. Understanding these factors is crucial for comprehending our study population, and is potentially instrumental in determining appropriate screening practices. For instance, the 2016 AAO guidelines suggest initiating annual monitoring earlier than five years of use if the daily dose/real body weight exceeds 5 mg/kg. Future research should explore the relationship between this major risk factor and hydroxychloroquine retinopathy screening practices.

In conclusion, this nationwide cohort study identified several demographic and clinical factors associated with HCQ retinopathy screening in South Korea. The findings highlight the influence of factors such as the level of care provided by prescribing and screening hospitals, the department of HCQ prescription, and the patient's underlying diagnosis. These findings can inform the development of targeted interventions and policies to improve the implementation of retinopathy screening guidelines, thereby optimizing the ocular safety of HCQ therapy in at-risk patients. Further research is warranted to validate these findings and to explore additional factors that may influence screening practices in other populations.

### Supplementary Information

Supplementary Tables.

## Data Availability

The datasets generated during and/or analyzed during the current study are available from the corresponding author on reasonable request.
