# Supplementary material for: Demographic and clinical characteristics associated with screening practices for hydroxychloroquine retinopathy
Source: Sci Rep. 2024 Jan 10;14:974. doi: 10.1038/s41598-024-51667-7 (PMC10782023; doi:10.1038/s41598-024-51667-7)
Supplement: Supplementary file 1 — Supplementary Tables. [file 41598_2024_51667_MOESM1_ESM.docx]

**Supplementary Tables**

**Supplementary Table 1.** Association of screening hospitals (levels of care) with baseline and monitoring examinations

| **Hospitals of baseline screening** | **Monitoring done in primary clinics** | **Monitoring done in referral centers** | **P-value** |
| --- | --- | --- | --- |
| Primary  Referral centers | 1555 (65.4%)  195 (1.5%) | 824 (34.6%)  12,903 (98.5%) | <0.001 |

**Supplementary Table 2.** Demographic and clinical characteristics between patients receiving additional modality (optical coherence tomography or automated visual fields) or not for baseline screening

| **Characteristics** | **No additional modality**  **(n=8057)** | **Additional modality* performed  (n=12,371)** | **P-value** |
| --- | --- | --- | --- |
| **Mean age** (years) | 48.6±13.8 | 45.3±15.5 | <0.001 |
| **Sex** |  |  |  |
| Male  Female | 1206 (40.7%)  6851 (39.2%) | 1755 (59.3%)  10,616 (60.8%) | 0.121 |
| **Residence** |  |  | <0.001 |
| Metropolitan area/large cities  Small cities/rural | 3943 (36.9%)  4114 (42.2%) | 6748 (63.1%)  5623 (57.8%) |  |
| **Medical specialties prescribing HCQ** |  |  |  |
| Rheumatology  Dermatology  Internal medicine other than rheumatology  Others | 4485 (35.6%)  250 (33.9%)  2516 (47.9%)  806 (44.0%) | 8117 (64.4%)  488 (66.1%)  2740 (52.1%)  1026 (56.0%) | <0.001 |
| **Hospitals of prescription** |  |  |  |
| Primary  Secondary/ tertiary | 1870 (54.4%)  6187 (36.4%) | 1569 (45.6%)  10,802 (63.6%) | <0.001 |
| **Hospitals of baseline screening** |  |  |  |
| Primary  Referral centers | 3181 (40.1%)  4876 (39.0%) | 4750 (59.9%)  621 (61.0%) | 0.120 |
| **Indications for HCQ use** |  |  |  |
| SLE  RA  Others | 1278 (26.2%)  5347 (46.0%)  1432 (36.4%) | 3608 (73.8%)  6265 (54.0%)  2498 (63.6%) | <0.001 |
| **Mean duration** (month) | 30.7±20.6 | 36.5±22.1 | <0.001 |
| **Mean daily dose** (mg) | 255.5±80.6 | 244.4±74.4 | <0.001 |

HCQ, hydroxychloroquine; SLE, systemic lupus erythematosus; RA, rheumatoid arthritis.

*Optical coherence tomography or automated visual fields.

**Supplementary Table 3**. Patterns of utilization for each of 4 screening modalities recommended by the 2016 AAO guideline for retinopathy monitoring in the overall monitored patients and in residence and screening hospital subgroups.

| **Screening modality** | **No. of performed (% among overall monitored patients)** | **Residence subgroups** | | **Screening hospitals** | |
| --- | --- | --- | --- | --- | --- |
|  |  | **Metropolitan/large cities,**  **No. of performed (%)** | **Small cities/rural,**  **No. of performed (%)** | **Primary clinics,**  **No. of performed (%)** | **Referral centers**  **No. of performed (%)** |
| OCT | 4310 (91.5%) | 2379 (91.7%) | 1931 (91.2%) | 1603 (91.5%) | 2707 (91.5%) |
| Visual fields | 3140 (66.7%) | 1892 (72.9%) | 1248 (59.0%) | 847 (48.3%) | 2293 (77.5%) |
| FAF | 1992 (42.3%) | 1395 (53.8%) | 597 (28.2%) | 208 (11.9%) | 1784 (60.3%) |
| mfERG | 192 (4.1%) | 102 (3.9%) | 90 (4.3%) | 25 (1.4%) | 167 (5.6%) |

OCT, optical coherence tomography; FAF, fundus autofluorescence; mfERG, multifocal electroretinogram

**Supplementary Table 4.** Interval between first and second monitoring examination and association with demographic and clinical characteristics

| **Characteristics** | **Within 1 year**  **(n=422)** | **1–2 years**  **(n=449)** | **2 years or longer**  **(n=120)** | **P-value** |
| --- | --- | --- | --- | --- |
| **Age** (years) | 42.6±16.0 | 40.3±13.9 | 42.2±14.8 | 0.071 |
| **Sex** |  |  |  |  |
| Male  Female | 51 (48.1%)  371 (41.9%) | 39 (36.8%)  410 (46.3%) | 16 (15.1%)  104 (11.8%) | 0.163 |
| **Residence** |  |  |  |  |
| Metropolitan area/large cities  Small cities/rural | 274 (42.6%)  148 (42.5%) | 296 (46.0%)  153 (44.0%) | 73 (11.4%)  47 (13.5%) | 0.583 |
| **Medical specialties prescribing HCQ** |  |  |  |  |
| Rheumatology  Dermatology  Internal medicine other than rheumatology  Others | 307 (42.5%)  12 (48.0%)  70 (42.4%)  33 (41.8%) | 334 (46.3%)  11 (44.0%)  70 (42.4%)  34 (43.0%) | 81 (11.2%)  2 (8.0%)  25 (15.2%)  12 (15.2%) | 0.753 |
| **Hospitals of prescription** |  |  |  |  |
| Primary  Referral centers | 25 (47.2%)  397 (42.3%) | 21 (39.6%)  428 (45.6%) | 7 (13.2%)  113 (12.1%) | 0.694 |
| **Indications for HCQ use** |  |  |  |  |
| SLE  RA  Others | 188 (45.3%)  153 (40.6%)  81 (40.7%) | 178 (42.9%)  173 (45.9%)  98 (49.3%) | 49 (11.8%)  51 (13.5%)  20 (10.1%) | 0.428 |
| **Mean duration** (month)  **Mean daily dose** (mg) | 93.9±19.2  242.1±68.6 | 98.7±18.7  232.3±65.2 | 108.4±19.0  241.9±62.9 | **<0.001**  0.073 |

HCQ, hydroxychloroquine; SLE, systemic lupus erythematosus; RA, rheumatoid arthritis.
